# Validation of a COVID-19 self-assessment tool for the prediction of COVID-19 in a primary health care setting in Egypt

**DOI:** 10.1017/S1463423621000736

**Published:** 2021-11-25

**Authors:** Ekram W. Abd El-Wahab, Mohammed Metwally

**Affiliations:** 1 Department of Tropical Health, High Institute of Public Health, Alexandria University, Alexandria, Egypt; 2 Gladstone Institutes, University of California, San Francisco, CA 94158, USA; 3 Department of Endemic and Infectious Diseases, Alexandria Fever Hospital, Alexandria, Egypt

**Keywords:** COVID-19, Egypt, primary health care, SARS-CoV-2, self-assessment, triage

## Abstract

**Background:**

As SARS-CoV-2 infection is sweeping the globe, early identification and timely management of infected patients will alleviate unmet health care demands and ultimately control of the disease. Remote COVID-19 self-assessment tools will offer a potential strategy for patient guidance on medical consultation versus home care without requiring direct attention from healthcare professionals.

**Objective(s):**

This study aimed to assess the validity and interrater reliability of the initial and modified versions of a COVID-19 self-assessment prediction tool introduced by the Egyptian Ministry of Health and Population (MoHP) early in the epidemic. The scoring tool was released for the public through media outlets for remote self-assessment of SARS-CoV-2 infection connecting patients with the appropriate level of care.

**Methods:**

We evaluated the initial score in the analysis of 818 consecutive cases presenting with symptoms suggesting COVID-19 in a single-primary health care clinic in Alexandria during the epidemic in Egypt (mid-February through July). Validity parameters, interrater agreement and accuracy of the score as a triage tool were calculated versus the COVID-19 polymerase chain reaction (PCR) test.

**Results:**

A total of 818 patients reporting symptoms potentially attributable to COVID-19 were enrolled. The initial tool correctly identified 296 of 390 COVID-19 PCR +ve cases (sensitivity = 75.9%, specificity = 42.3%, positive predictive value = 54.5%, negative predictive value = 65.8%). The modified versions of the MoHP triage score yielded comparable results albeit with a better accuracy during the late epidemic phase. Recent history of travel [OR (95% CI) = 12.1 (5.0–29.4)] and being a health care worker [OR (95% CI) = 5.8 (2.8–11.9)] were major predictors of SARS-CoV-2 infection in early and late epidemic phases, respectively. On the other hand, direct contact with a respiratory infection case increased the risk of infection by three folds throughout the epidemic period.

**Conclusion:**

The tested score has a sufficient predictive value and potential as a triage tool in primary health care settings. Updated implementation of this home-grown tool will improve COVID-19 response at the primary health care level.

## Introduction

The world is facing a devastating pandemic of a novel coronavirus disease (COVID-19), caused by severe acute respiratory syndrome coronavirus 2 (SARS-CoV-2) (Singhal, 2020). The virus emerged and the outbreak was first identified in the disease epicentre in Wuhan, Hubei province of China, in late November 2019. The World Health Organization (WHO) 2020 declared the outbreak to be a Public Health Emergency of International Concern (PHEIC) on 30th January 2020 and recognized it as a pandemic on 11th March 2020 (World Health Organization [WHO], 2020a).

**Figure 1.**
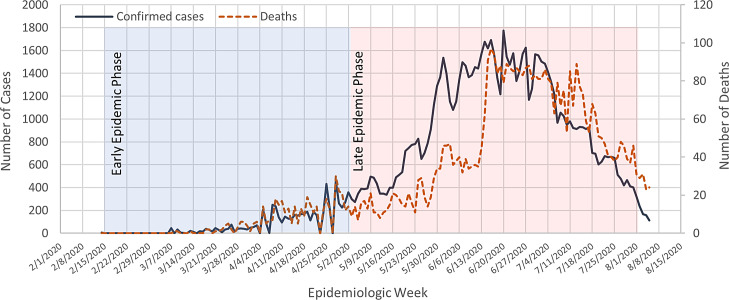
COVID-19 confirmed cases and deaths throughout the epidemic phases in Egypt

As of August 6th 2020, a total of 18,354,342 confirmed cases of COVID-19 have been reported in 213 countries and territories, resulting in a death toll of 696 147. For instance, more than 11,302,021 people have since recovered (World Health Organization [WHO], 2020a). Egypt has been experiencing widespread transmission of the virus in the community for several months, with an increasing number of COVID-19 outbreaks in healthcare facilities (World Health Organization [WHO], 2020a) (Figure 1).

With the rapidly increasing numbers of infections, COVID-19 has become a considerable strain for all health care settings. So far, we lack a rapid point of care test for SARS-CoV-2, and the diagnosis of COVID-19 is limited by the use of reverse transcription polymerase chain reaction (RT-PCR) which is not widely available or more time-consuming, thus not practicable at primary health care (PHC) settings. Moreover, expanding testing supplies and capabilities is lagging behind what is needed to curb such unprecedented pandemic scale. Clinical prediction scores support the assessment of patients in the PHC setting to determine the need for further diagnostic and therapeutic steps (Sheridan, 2020).

Early in the epidemic, the Egyptian Ministry of Health and Population (MoHP) introduced a self-assessment score as remote care solution to support emergency services in the fight against COVID-19 (Figure S1). It combines specific clinical and epidemiological features to determine an individual’s risk of having COVID-19. The MoHP has released the score for the public in the local language as a free online tool supported by a disease control hotline. Users can access the tool through media outlets and the MoHP official website. Based on their input, patients can have telephone consultations with a healthcare professional to determine the need for testing or be guided for home self-care (The Egyptian Ministry of Health and Population (MoHP), 2020a).

We evaluated the MoHP COVID-19 prediction score in an analysis of 818 consecutive patients with suspected COVID-19 from mid-February until mid-July 2020.

## Methods

### Study setting, design and population

The study was conducted at a PHC clinic in Alexandria between February and July 2020. Patients presenting with acute respiratory symptoms were enrolled consecutively and evaluated in order to determine the initial features which may help to distinguish probable COVID-19 cases from other respiratory problems. All patients were evaluated with chest imaging and blood testing. Detection of SARS-CoV-2 in nasopharyngeal swabs by RT-PCR test was done at a central reference laboratory.

Due to variations of the disease occurrence over time, study sample size estimations were not performed, and alternatively, sample size was determined by practical convenience.

The case definition of a COVID-19 suspected or confirmed case was set by the MoHP and was based on the encounter of elements of the epidemiological history as well as clinical symptoms (Table 1) (The Egyptian Ministry of Health and Population (MoHP), 2020b).

**Table 1.** Case definition of a COVID-19 suspected or confirmed case

Suspected COVID-19 case	Confirmed COVID-19 case
**Epidemiological history**	A suspected COVID-19 case that has been confirmed to have SARS-CoV-2 infection by RT-PCR test (+ve RNA SARS-CoV-2)
Travel to or residence in Wuhan in the last 14 days prior to symptom onset	
Recent travel history to high-risk area (traveled or resided in any community or country where confirmed Covid-19 cases existed, within 14 days before symptoms appear)	
Contact with a confirmed or suspected case of SARS-CoV-2 infection in the last 14 days prior to symptom onset	
Physician/health care worker discretion	
Contact with a patient who has fever or respiratory symptoms or from a community with confirmed cases reported within the last 14 days	
**Clinical manifestations**	
Fever and/or respiratory infection, or with normal/decreased white blood cell counts and normal/decreased lymphocyte counts	
Symptoms of respiratory illness (cough, fever, flu-like symptoms)	
Imaging characteristics (CT scan or chest X ray)	
Aggressive disease onset (Severe Acute Respiratory Infection (SARI) with no other obvious cause)	

### Scoring tools

To assess the validity of the MoHP self-assessment score, we applied it for all the enrolled suspected COVID-19 patients and tested its performance versus the finally obtained diagnosis. The score includes nine questions with each criterion scored with a different number of points. Q1: Fever (≥ 38.0°C) (2 points), Q2: Severe cough (2 points), Q3: Severe sore throat (1 point), Q4: Vomiting or diarrhea (0 point), Q5: Chronic disease (diabetes mellitus, hypertension, ischemic heart disease, renal disease, liver disease, autoimmune disease, etc….) (1 point), Q6: Travel in or outside Egypt (Sharm Elshiek, or Europe, China or any country) (5 points), Q7: Direct contact to a respiratory infection case (care, face to face) (4 points), Q8: Visiting health care place that had received confirmed case (3 points), Q9: Being a health care worker (HCW) (2 points).

A numeric score is calculated by counting the number of criteria met at the initial presentation (score range 0–20 points). A cut-off of 6 points and more was highly suggestive for COVID-of infection and was set for calling the disease control hotline, whereas a score of 4 necessitated home isolation and a score of 5 was required before consulting a health care physician.

### Statistical analysis

The collected data were reviewed for accuracy and integrity and entered into computer software. For all statistical analyses, we used MS Excel 2016 and a statistical software package (IBM® SPSS^®^ Statistics Base, IBM Corp: Armonk, NY; version 21.0). We afterward summarized all variables. Continuous variables are presented as the mean ± standard deviation (SD). Categorical variables are expressed as numbers with proportions, n (%). Chi-Square test, Fisher’s exact tests, Student’s *t*-tests, multivariate logistic regression and ROC curve analyses were performed for group comparisons.

The association between patient’s variables (clinical and sociodemographic) and having a SARS-CoV-2 positive PCR test was examined using a univariate analysis. We run this step three times to infer the association in early epidemic phase, late epidemic phase and throughout the epidemic. The variables associated with SARS-CoV-2 positive PCR test at the *P* < 0.05 significance level on univariate analyses and deemed potentially useful for the clinico-epidemiological prediction of the disease were selected for multivariable analysis. The final list of eligible variables differed in relation to the epidemic phase. The selected variables were entered as covariates to develop a multivariable logistic regression equation by backward (Wald) step-wise elimination, with SARS-CoV-2 positive PCR test being the outcome variable.

### Testing the validity and accuracy of the prediction scores

After testing the MoHP scoring system (thereafter score 1), we created two new scores named ‘score 2’ and ‘score 3’. In score 2, vomiting or diarrhea symptoms in Q4 worth 1 point, and score 3 additionally included patient age [20 years (0 point), 20–35 (1 point), 36–55 (2 points) and 56–99 (3 points)] and smoking status [non-smoker/ex-smoker (0 point) and current smoker (1 point)]. The aggregate of these weighted variables was expressed as a total score (prediction index) for each patient individually. A receiver operating characteristic (ROC) curve was plotted with the total score as the test variable and SARS-CoV-2 PCR test as the state variable. The area under the ROC curve (AUC) was used to assess its overall predictive performance, sensitivity, specificity, positive and negative predictive values. We identified the optimal cut-off (Youden index) of the total score from the ROC curve as the point corresponding to the best trade-off between sensitivity and specificity.

We assessed the goodness-of-fit of all the prediction models using the Hosmer and Lemeshow test.

## Results

### Characteristics of the study cohort

Of a total of 818 suspected COVID-19 patients presenting with acute respiratory symptoms were enrolled in the study, 390 (47.7%) were confirmed to have SARS-CoV-2 by RT-PCR. The mean age of SARS-CoV-2 positive subjects was 43.0 ± 17.0 years, with women being more affected than men (53.8% vs. 46.8% respectively) (Table 2). The total scores met at presentation differed significantly (*t* = −8.5, *P* < 0.001) between SARS-CoV-2 positive (mean ± SD = 9.3 ± 3.9, standard error = 0.2) and SARS-CoV-2 negative (mean ± SD = 7.1 ± 3.2, standard error = 0.15) patients (Table 2).

**Table 2.** Clinico-epidemiological characteristics of the enrolled COVID-19 suspected cases

		Total (n = 818)		COVID-19 PCR Test				*p*
				Negative (n = 428)		Positive (n = 390)		
		No.	%	No.	%	No.	%	
Age (years)	<20	58	7.1	34	7.9	24	6.2	.007
	20–35	276	33.7	165	38.6	111	28.5	
	35–55	296	36.2	141	32.9	155	39.7	
	55–100+	188	23.0	88	20.6	100	25.6	
Mean ± SD [Range (years)]		41.3 ± 17.3 (0.3–99)		39.7 ± 17.5		43.0 ± 17.0		*t* = –2.7, *p* = .007
Epidemic phase	Early	449	54.9	208	48.6	241	61.8	
	Late	369	45.1	220	51.4	149	38.2	<0.001
Sex	Male	367	44.9	187	43.7	180	46.2	
	Female	451	55.1	241	56.3	210	53.8	.479
Smoking	Never	614	75.1	329	76.9	285	73.1	
	Smoker	202	24.7	98	22.9	104	26.7	.456
	Ex-Smoker	2	0.2	1	0.2	1	0.3	
Q1: Fever (Temperature >38 °C)	No	151	18.5	76	17.8	75	19.2	.587
	Yes	667	81.5	352	82.2	315	80.8	
Q2: Severe cough	No	212	25.9	86	20.1	126	32.3	<0.001
	Yes	605	74.0	341	79.9	264	67.7	
Q3: Severe sore throat	No	377	46.1	208	48.6	169	43.3	.131
	Yes	441	53.9	220	51.4	221	56.7	
Q4: Vomiting or diarrhea	No	600	73.3	323	75.5	277	71.0	.151
	Yes	218	26.7	105	24.5	113	29.0	
Q5: Chronic disease (DM, HT, IHD, renal disease, liver disease, autoimmune disease, etc….)	No	560	68.5	287	67.1	273	70.0	.365
	Yes	258	31.5	141	32.9	117	30.0	
Q6: Travel in or outside Egypt (Sharm Elshiek, or Europe, China or any country)	No	702	85.8	417	97.4	285	73.1	<0.001
	Yes	116	14.2	11	2.6	105	26.9	
Q7: Direct contact to a respiratory infection case (care, face-to-face)	No	255	31.2	183	42.8	72	18.5	<0.001
	Yes	563	68.8	245	57.2	318	81.5	
Q8: Visiting health care place that had received confirmed case	No	700	85.6	369	86.2	331	84.9	.585
	Yes	118	14.4	59	13.8	59	15.1	
Q9: Being a health care worker	No	704	86.1	384	89.7	320	82.1	.002
	Yes	114	13.9	44	10.3	70	17.9	
Total symptoms’ score [mean ± SD]		8.1 ± 3.7		7.1 ± 3.2		9.3 ± 3.9		*t* = –8.5, *P* < 0.001

DM = diabetes mellitus; HT = hypertension; IHD = ischemic heart disease; PCR = polymerase chain reaction; SD = standard deviation.

*t*, *t* value for student *t*-test.

*p* significant at < 0.05.

### Performance of the prediction scores

**Figure 2.**
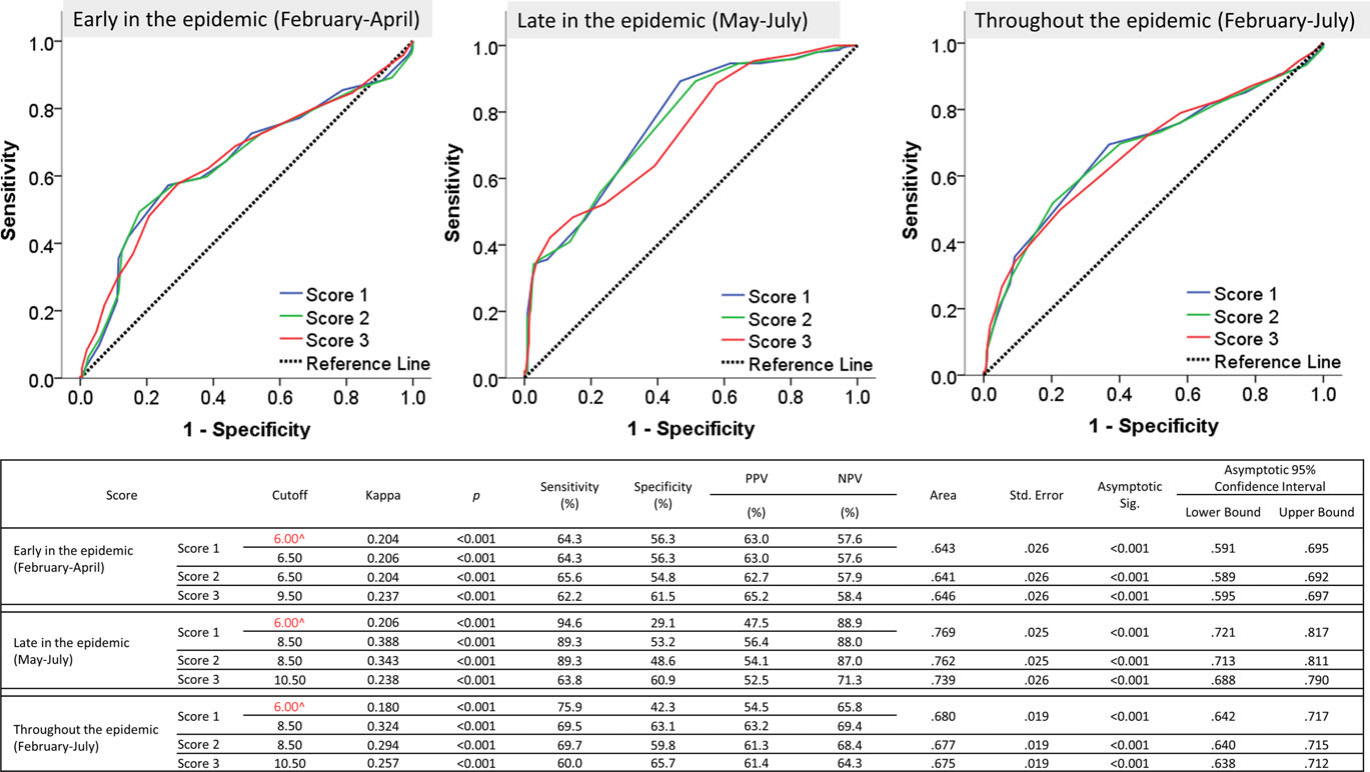
Performance and validation of the different scores in the prediction of COVID-19 versus the reference PCR test ^initial cutoff point set by the MOHP for Suspected SARS-CoV-2 infection

The validation sample was split up in an early and late phase of the epidemic as driven by the results of data analysis. The performance of the three tested scores (1, 2 or 3) was comparable regarding the prediction of SARS-CoV-2 infection when tested early in the epidemic (February–April), late in the epidemic (May–July) as well as throughout the epidemic period (February–July) (Figure 2). A cut-off of 6.0 points met in the initial score (score 1) was associated with correct RT-PCR-based SARS-CoV-2 detection in nasopharyngeal swabs and presence of symptomatic COVID-19 in 296 out of 390 patients (sensitivity = 75.9%, specificity = 42.3%, positive predictive value = 54.5%, negative predictive value = 65.8%), whereas lower scores were associated with other respiratory conditions (bronchitis, pneumonia, common cold). When we adopted another cut-off point (8.5) with a better trade-off between sensitivity and specificity, more accurate score performance was obtained (sensitivity = 69.5%, specificity = 63.1%, positive predictive value = 63.2%, negative predictive value = 69.4%). Collectively, the tested prediction scores had a sensitivity range of 60.0%–89.3%, a specificity range of 48.6%–65.7% and corresponding positive and negative predictive value ranges of 52.5–65.2% and 57.6%–88.0%, respectively. Likewise, the interrater reliability of the initial and the derived scores versus the diagnostic PCR test used for confirming the diagnosis of COVID-19 showed minimal degrees of agreement, with kappa values ranging between 0.204 and 0.388 (*P* < 0.001). The AUC ranged between 0.641 (95% CI = 0.589–0.692) and 0.769 (95% CI = 0.721–0.817), (*P* < 0.001). Overall, the performance of the triage scores was more accurate in predicting SARS-CoV-2 infection later in the epidemic (Figure 2).

All the derived models showed appropriate goodness-of-fit (Hosmer and Lemeshow *P* > 0.05).

### Predictors of COVID-19 infection

On univariate analysis, the variables associated with the presence of COVID-19 infection in patients presenting with acute respiratory symptoms at the *P* < 0.05 significance level differed according to the epidemic phase (Tables 2 and 3). Step-wise elimination generated epidemic phase wise predictor variables in the final multivariable equations. Early in the epidemic, history of travel in or outside Egypt [OR (95% CI) = 12.1 (5.0–29.4)] and direct contact with a respiratory infection case [OR (95% CI) = 3.5 (2.1–5.6)] were strong predictors of COVID-19 infection. On the other hand, having severe cough [OR (95% CI) = 0.29 (0.18–0.48)] or visiting health care place that had received COVID-19 confirmed cases [OR (95% CI) = 0.73 (0.19–0.74)] reduced the likelihood of having COVID-19. Later in the epidemic, having fever [OR (95% CI) = 28.1 (3.7–214.0)], severe cough [OR (95% CI) = 3.7 (1.4–9.8)], severe sore throat [OR (95% CI) = 2.9 (1.5–5.8)], direct contact with a respiratory infection case [OR (95% CI) = 4.3 (1.7–10.9)], and being a HCW [OR (95% CI) = 5.8 (2.8–11.9)] increased the odds of having COVID-19 infection. Throughout the epidemic period, older age [OR (95% CI) = 1.01 (1.0–1.02)], history of travel [OR (95% CI) = 10.6 (5.5–20.4)], direct contact with a respiratory infection case [OR (95% CI) = 3.1 (2.1–4.4)], and being a HCW [OR (95% CI) = 2.2 (1.4–3.4)] increased the risk of having SARS-CoV-2 infection, whereas having severe cough was identified as a negative disease predictor [OR (95% CI) = 0.50 (0.34–0.73)] (Table 4).

**Table 3.** Clinico-epidemiological characteristics of the enrolled COVID-19 suspected cases in early versus late epidemic phases

		COVID-19 PCR test									
		Early in the epidemic (n = 449)				*p*	Late in the epidemic (n = 369)				*p*
		Negative (n = 208)		Positive (n = 241)			Negative (n = 220)		Positive (n = 149)		
		No.	%	No.	%		No.	%	No.	%	
Age (years)	<20	11	5.3	13	5.4	.013	23	10.5	11	7.4	.529
	20–35	75	36.1	54	22.4		90	40.9	57	38.3	
	35–55	68	32.7	104	43.2		73	33.2	51	34.2	
	55–100+	54	26.0	70	29.0		34	15.5	30	20.1	
Mean ± SD		42.5 ± 16.7		45.2 ± 15.8		*t* = –1.8, *p* = .080	37.1 ± 17.6		39.4 ± 18.2		*t* = –1.2, *p* = .22
Sex	Male	103	49.5	130	53.9	.350	84	38.2	50	33.6	.365
	Female	105	50.5	111	46.1		136	61.8	99	66.4	
Smoking	Never	157	75.5	172	71.4	.604	172	78.2	113	75.8	.598
	Smoker	50	24.0	68	28.2		48	21.8	36	24.2	
	Ex-Smoker	1	0.5	1	0.4		0	0.0	0	0.0	
Q1: Fever (Temperature >38°C)	No	39	18.8	74	30.7	.004	37	16.8	1	0.7	<0.001
	Yes	169	81.3	167	69.3		183	83.2	148	99.3	
Q2: Severe cough	No	52	25.1	119	49.4	<0.001	34	15.5	7	4.7	.001
	Yes	155	74.9	122	50.6		186	84.5	142	95.3	
Q3: Severe sore throat	No	119	57.2	153	63.5	.175	89	40.5	16	10.7	<0.001
	Yes	89	42.8	88	36.5		131	59.5	133	89.3	
Q4: Vomiting or diarrhea	No	159	76.4	183	75.9	.900	164	74.5	94	63.1	.019
	Yes	49	23.6	58	24.1		56	25.5	55	36.9	
Q5: Chronic disease (DM, HT, IHD, renal disease, liver disease, autoimmune disease, etc….)	No	137	65.9	175	72.6	.121	150	68.2	98	65.8	.628
	Yes	71	34.1	66	27.4		70	31.8	51	34.2	
Q6: Travel in or outside Egypt (Sharm Elshiek, or Europe, China or any country)	No	202	97.1	148	61.4	<0.001	215	97.7	137	91.9	.009
	Yes	6	2.9	93	38.6		5	2.3	12	8.1	
Q7: Direct contact to a respiratory infection case (care, face-to-face)	No	125	60.1	65	27.0	<0.001	58	26.4	7	4.7	<0.001
	Yes	83	39.9	176	73.0		162	73.6	142	95.3	
Q8: Visiting health care place that had received a confirmed case	No	165	79.3	226	93.8	<0.001	204	92.7	105	70.5	<0.001
	Yes	43	20.7	15	6.2		16	7.3	44	29.5	
Q9: Being a health care worker	No	186	89.4	216	89.6	.944	198	90.0	104	69.8	<0.001
	Yes	22	10.6	25	10.4		22	10.0	45	30.2	

DM = diabetes mellitus; HT = hypertension; IHD = ischemic heart disease; PCR = polymerase chain reaction; SD = standard deviation.

*t*, *t* value for student *t*-test.

*p* significant at < 0.05.

**Table 4.** Predictors of SARS-CoV-2 infection in early versus late epidemic phases

Epidemic phase	Backward Stepwise (Wald) Logistic Regression	Variables	B	S.E.	Wald	df	Sig.	Exp(B)	95% C.I. for EXP(B)	
									Lower Limit	Upper Limit
Early in the epidemic (February–April)	Step 2	Fever (temperature >38 °C)	−.52	.27	3.7	1	.054	.60	.35	1.0
		Severe cough	−1.23	.25	24.6	1	<0.001	.29	.18	.48
		Travel in or outside Egypt	2.49	.45	30.1	1	<0.001	12.1	5.0	29.4
		Direct contact with a respiratory infection case	1.24	.24	26.1	1	<0.001	3.5	2.1	5.6
		Visiting health care place that had received a confirmed case	−.99	.35	7.9	1	.005	.37	.19	.74
		Constant	.40	.28	2.0	1	.161	1.5		
Late in the epidemic (May–July)	Step 4	Fever (temperature >38°C)	3.34	1.04	10.4	1	.001	28.1	3.7	214.0
		Severe cough	1.31	.49	7.0	1	.008	3.7	1.4	9.8
		Severe sore throat	1.07	.35	9.1	1	.003	2.9	1.5	5.8
		Direct contact with a respiratory infection case	1.46	.47	9.6	1	.002	4.3	1.7	10.9
		Being a health care worker	1.76	.37	22.9	1	<0.001	5.8	2.8	11.9
		Constant	−7.14	1.23	34.0	1	<0.001	.00		
Throughout the epidemic (February–July)	Step 1	Age	.01	.00	7.7	1	.006	1.01	1.00	1.02
		Severe cough	−.69	.19	13.0	1	<0.001	.50	.34	.73
		Travel in or outside Egypt	2.36	.33	49.9	1	<0.001	10.6	5.5	20.4
		Direct contact with a respiratory infection case	1.12	.18	37.8	1	<0.001	3.1	2.1	4.4
		Being a health care worker	.80	.22	13.0	1	<0.001	2.2	1.4	3.4
		Constant	−1.25	.28	19.3	1	<0.001	.29		

C.I. = Confidence interval; df = degree of freedom.

*p* significant at < 0.05.

## Discussion

Based on the evaluation of the initial data of 818 patients presenting with acute respiratory symptoms, we assessed the diagnostic performance of the MoHP score as a simple self-assessment tool for COVID-19 prediction. The score combines consistently available clinico-epidemiological elements and thus it is ideal for triage in PHC settings.

During the COVID-19 pandemic and under severe time constraints, the MoHP score was released for the public as a virtual assessment tool for selecting symptomatic COVID-19 cases from rather unspecific general or respiratory symptoms and prioritize those with a higher epidemiological probability of the disease for further medical attention. Based on personal symptoms, exposure and risk factors, individuals can find the right care by either assurance, home self-care, consulting a physician or immediately calling the disease control centre to get a SARS-CoV-2 swab. However, since the tool was created very early in the epidemic with little accumulation of relevant clinical, there was no clear or definitive data available regarding the most specific frequent symptoms encountered in SARS-CoV-2 infection, the tool lacked some important symptoms frequently encountered by COVID-19 patients including altered taste or smell, headache, confusion, difficult breathing, myalgia and fatigue (Harapan et al., 2020, Lüers et al., 2020, Cummings et al., 2020). It is worth noting that difficult breathing may have not been considered in the scoring system because symptoms usually begin as mild in all patients with occasionally dyspnea manifesting in severe cases (Cascella et al., 2020). Although the score included gastrointestinal symptoms, a common presentation in COVID-19 disease (Hajifathalian et al., 2020, Goyal et al., 2020), it ignored its scoring. However, when a history of vomiting or diarrhea worth 1 point in our proposed score 2 and score 3, this did not result in an appreciable change in the performance of the initial score.

Although relying on respiratory symptoms was not appropriate to identify individuals with COVID-19 early in the disease, epidemiological risk factors such as travel history and direct exposure to a symptomatic case were strong predictors of SARS-CoV-2 infection. This suggests that the clinical differentiation of COVID-19 from other respiratory illnesses at the early stages of the disease is limited. One reason could be that the symptoms included in the scoring system were relatively unusual although they appear to be much more common in other countries, a difference which may reflect geographic variation or differential reporting. Another possibility is that travelers returning from the disease epicentres in Europe and the hardest-hit regions (for our study cohort; China, Italy, Spain, Germany, USA and KSA) at that time were more attentive of their symptoms and sought medical advice immediately before they develop overt symptoms.

Late in the epidemic, the risk attributable to travel disappeared from the model which reflects international travel bans during this period. Conversely, the disease started to affect more HCWs. The risk increased by 5.8-fold compared to the early epidemic phase (30.2% vs. 10.4% respectively, *P* < 0.001). This could also reflect that they had been tested earlier and more frequently.

From the beginning of the epidemic, older age has been identified as an important risk factor for disease susceptibility, severity and outcome as explained by the age-related abundance of the angiotensin converting enzyme 2 (ACE2) receptors that may facilitate viral invasion (Guan et al., 2020, Huang et al., 2020, Livingston and Bucher, 2020, McMichael et al., 2020). In the present report, advancing age was associated with one-fold higher odds of being COVID-19 positive throughout the epidemic period. Nevertheless, including patient age in score 3 did not improve its accuracy in predicting SARS-CoV-2 infection.

Likewise, smoking neither increased the risk of COVID-19 nor improved the accuracy of the initial prediction score. Indeed, smoking as a risk factor for SARS-CoV-2 infection is under discussion although pooled analysis linked it to increased disease severity and death in hospitalized COVID-19 patients (World Health Organization [WHO], 2020b, Grundy et al., 2020, Engin et al., 2020). Furthermore, although COVID-19 infections are relatively more likely to be seen in men (Park, 2020), the risk was comparable in both sexes throughout the epidemic phases. So, it may be difficult to identify who will more likely develop the illness by using some established risk factors such as age, sex, smoking and comorbidities. Nevertheless, as the COVID-19 pandemic evolves, the accumulation of relevant clinical and other health information will likely lead to the development of a more robust risk prediction model and increase the success of assessment strategies to support medical decision making.

The structured questions in the MoHP score did not include a focus on change in symptoms or assessing clinical deterioration in mild disease, which might have a role in the clinical assessment.

Given its modest accuracy and to avoid misclassifying patients, the tested prediction tool could be considered as one component within a broader clinical evaluation of patients presenting with acute respiratory symptoms since for instance, it gives attention to a trajectory of symptoms and patient characteristics such as age, exposure and comorbid conditions.

So far, there is no reliable risk assessment tool for predicting COVID-19 in daily practice. Wynants et al. (2020) conducted a systematic review of several COVID-19 prediction models ranging from rule-based scoring systems to advanced machine learning models that have been rapidly proposed and published in response to the pandemic. Most of these models were found of limited quality, at high risk of bias and inadequate in making precise predictions about disease risk and outcomes (Wynants et al., 2020). It is worth noting that the currently available data suggest suboptimal test performance for the gold standard test “RT-PCR” since it detected only the SARS-CoV-2 virus in 63% of nasal swabs and 32% of pharyngeal swabs in patients with the known disease (Wang et al., 2020).

The proposed tool is well-suited to carry out a preliminary assessment of suspected patients and help them to get timely treatment and quarantine suggestion. Since COVID-19 is changing the face of health care delivery, the adaptation of this tool could be useful in virtual primary care or community settings. The predictors identified in included models should be considered as candidate predictors for new models. However, to accommodate an evolving COVID-19 pandemic, the proposed model will need to be recalibrated and refit over time. Widespread dissemination, tracking of its utilization, and direct integration with the digital health record can further improve its utility.

This type of self-assessment tool provides immediate benefit to the patients and health care providers as we face anticipated increased demand and limited resources. They undoubtedly have the potential to (i) facilitate patient triage and preventing unnecessary in-person visits during the COVID-19 pandemic to prevent patient exposure to pathogens in their route to clinic visits and in waiting rooms, (ii) avoid unnecessary testing of a large proportion of patients who are indeed COVID negative, (iii) reduce personal protective use by clinic staff and liberate front-line staff to care for sicker patients. However, it remains unclear how many patients might be harmed if the tool falsely predicted that self-care was sufficient, and thus limit its use in the context of a pandemic (Jehi et al., 2020, Wynants et al., 2020).

## Conclusion

An updated implementation of this home-grown tool holds promise and provides new insights into the rapid diagnosis, the timely isolation and treatment of COVID-19, which are currently the known most effective response. Given its potential implications in terms of global health and in improving the COVID-19 response at the PHC level, the proposed tool could be shared with other health organizations and authorities in other countries. The tool can be deployed in a web-based or smartphone contexts as an interactive clinical assessment digital tool or a Self-Checker paired with resources and recommended actions. In this regard, it can be customized to fit the needs of both the individual and the community. Based on user’s responses, the tool can provide valuable information on seeking medical care specific to his area. Moreover, it can track geographic trends by creating heat map zones in cases when users share their zip codes. A further credible use of this tool is to provide passengers entering from risky countries to check their symptoms daily for 14 days. This will ultimately help in combating the spread of the disease globally.

## Limitations of the study

The major limitation of this work is the single-centre evaluation of only a limited number of patients representing a convenience sample. Early in the epidemic, the MoHP score was introduced to predict whether a user of the tool should seek a particular level of care. However, we evaluated how this model predicts the probability of a current COVID-19 diagnosis. Indeed, the introduced MoHP tool predicts three triage levels (the probability of COVID-19 infection, the requirement for home isolation and consultation). Yet, these outcomes were not evaluated in the main analysis, which revolved around the distinction between COVID-19 positive and negative outcomes only. It is noteworthy that the introduced self-assessment tool is supposed to be used by individuals in the general population, while we evaluated its performance in individuals that have already presented themselves at PHC. Thus, the selected individuals do not seem to match the research question of interest. The fact that the tool was not evaluated in the target population might result in a bias (self-selection bias) that cannot be corrected for the analysis phase of this study. We must also acknowledge that the clinical motivation for the different cut-off was not described due to limited access to the baseline data used for creating the score. Moreover, the application of the proposed scores on the identification of COVID-19 cases with atypical symptoms was not confirmed which limits their use in clinical practice.

While the preliminary results were promising, it is obvious that the MoHP score needs to be thoroughly updated and validated in further large-scale studies conducted across different populations in larger cohorts of patients to exploit our findings and gain more reliable data regarding its diagnostic yield. For instance, the score may be used for the assessment of patients with suspected COVID-19, but with some caution undertaken in this context. We did not keep track of the disease outcomes in the study cohort and therefore we could not assess the appropriateness of our tool’s recommendations. Like the majority of the currently available COVID-19 diagnostic models, we used viral nucleic acid test results as the gold standard, which may have unacceptable false negative rates (Wynants et al., 2020). The strengths are its simplicity, immediate availability as well as wide applicability due to simple components.

## Data Availability

All data are fully available without restriction by the corresponding author at ekram.wassim@alexu.edu.eg the and through the public data repository “Harvard Dataverse” at https://dataverse.harvard.edu/dataverse

## Supplementary material

For supplementary material accompanying this paper visit http://dx.doi.org/10.1017/S1463423621000736. click here to view supplementary material
