# Supplementary figures and images for: Validation of a COVID-19 self-assessment tool for the prediction of COVID-19 in a primary health care setting in Egypt
Source: Prim Health Care Res Dev. 2021 Nov 25;22:e75. doi: 10.1017/S1463423621000736 (PMC8628560; doi:10.1017/S1463423621000736)

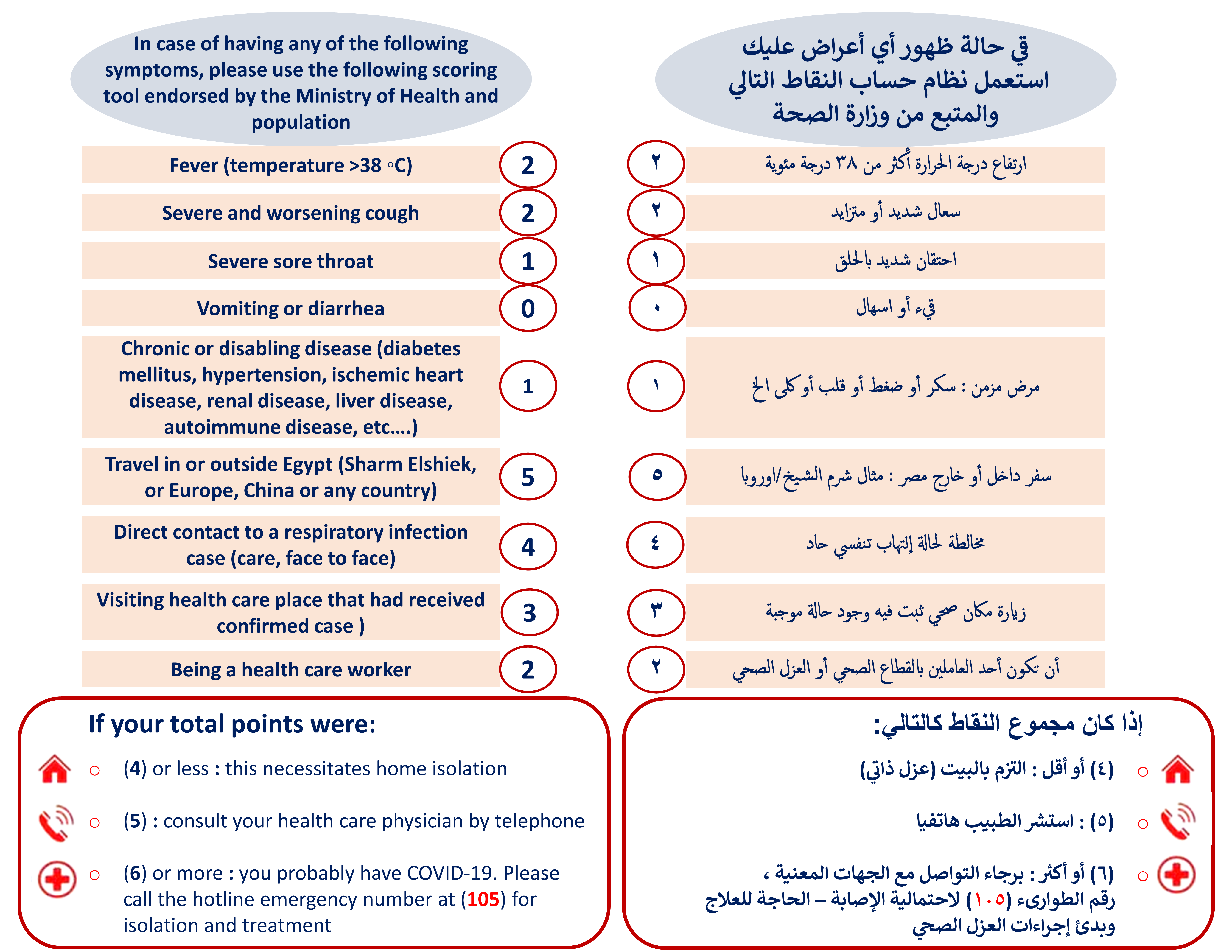

Supplement: Supplementary file 1 [file S1463423621000736sup001.tif]
